# Cross-Culture Development and Validation of a Children’s Psychological Richness Questionnaire Using Artificial Neural Network Analysis

**DOI:** 10.3390/bs16060931

**Published:** 2026-06-05

**Authors:** Boshra A. Arnout

**Affiliations:** 1 Center for Human and Philosophical Studies, King Khalid University, Abha 61421, Saudi Arabia; beahmad@kku.edu.sa or prof.arnout74@gmail.com; 2 Department of Psychology, College of Education, King Khalid University, Abha 61421, Saudi Arabia; 3 Department of Psychology, College of Arts, Zagazig University, Zagazig 44519, Egypt

**Keywords:** psychological richness, artificial neural networks, factorial structure, children

## Abstract

This study aimed to develop and validate a children’s psychological richness questionnaire across cultural contexts using a descriptive approach. The study sample consisted of 1041 children aged 10–17 years (12.94 ± 2.14), including 440 Saudi Arabian and 601 Egyptian participants. The results indicated a general one-factor structure in both the Saudi sample and the total sample, whereas two main factors emerged in the Egyptian sample. Moreover, the exploratory factor analysis showed that the two-factor model provided a better fit than the general one-factor, as demonstrated by the Akaike and Bayesian information indices, reflecting the model’s efficiency in representing the psychological structure of children’s psychological richness. Additionally, the artificial neural network analysis identified the most central and influential items within each cultural context. The findings also revealed that the questionnaire has a high degree of internal consistency. Furthermore, the results showed no significant differences in psychological richness between children in the Saudi and Egyptian contexts.

## 1. Introduction and Theoretical Background

Childhood is a crucial stage in human development, during which the biological, psychological, and social foundations of personality are established. It represents a period of “initial formation”, in which the surrounding environment and early experiences play a significant role in shaping mental and emotional capacities. Environmental and experimental factors during early life influence the level of psychological richness (PR) that children possess at this stage, and this level, in turn, affects their development in subsequent stages of life. From a positive psychology perspective, assessing and enhancing PR in childhood can contribute to more balanced and stable development in adolescence and adulthood, serving as a long-term investment in positive mental health and personal success.

Piaget (1936/1952) considered childhood to be the most critical stage in the development of cognitive processes, whereas Erikson (1963) emphasized that self-confidence and identity begin to form in the early years through the child’s interactions with parents and society. Therefore, the quality of care, emotional support, and cognitive stimulation during this stage have long-term effects on psychological well-being, life success, and social skills in later life. Consequently, childhood has emerged in the psychological literature (Shonkoff & Phillips, 2000) as the most crucial stage in shaping a person’s destiny, not merely a period of physical growth or chronological age.

Psychological richness represents a unique route to well-being distinct from both hedonic and eudaimonic approaches. Whereas hedonic well-being centers on pleasure and positive emotions, and eudaimonia well-being emphasizes purpose and self-realization, psychological richness highlights the value of varied intellectually stimulating experiences marked by novelty, challenge, and cognitive engagement (Oishi et al., 2020, 2025).

PR is a relatively recent concept in positive psychology, representing a distinct dimension of psychological well-being that complements both happiness and meaning. It is characterized by a diversity of experiences, profound cognitive shifts, and ongoing personal development (Besser & Oishi, 2020; Mauro et al., 2025; Oishi et al., 2019b; Oishi & Westgate, 2022). Evidence indicates that individuals with psychologically rich lives tend to exhibit higher levels of openness and curiosity, and often experience complex emotional states that combine positive and negative feelings simultaneously, without this being associated with increased levels of depression or other psychological disorders (Oishi et al., 2019b). Moreover, unusual experiences or novel experiences, such as studying in new cultural contexts, are associated with significant increases in PR, while their impact on indicators of happiness or meaning remains limited, highlighting the specificity of this concept and its importance in psychological assessment (Oishi et al., 2020).

Given that many—and perhaps most—psychological constructs cannot be directly observed or measured, such as the concept of “PR in children”, there remains a fundamental need for highly reliable and validated measures of underlying constructs. Therefore, developing psychometric instruments with strong measurement properties and validity across multiple cultural contexts is a pressing need in modern psychology. The theoretical literature emphasizes the importance of testing factorial structure and establishing construct validity and reliability to ensure accurate assessment in diverse settings (Clark & Watson, 2019; DeVellis, 2016; Dwyer et al., 2018; Putnick & Bornstein, 2016).

Oishi et al. (2019a) developed the first PR questionnaire for individuals aged 18 and older in the United States. Numerous studies have examined the factor structure and reliability of this questionnaire across diverse cultural contexts. In their study, Oishi et al. (2019b) demonstrated that the factor structure of PR is unidimensional and exhibits good fit indices of conformity in samples from the United States and India. Similarly, Liu et al. (2024) found that the PR questionnaire developed by Oishi et al. (2019a) demonstrated good validity and reliability in the Chinese context, and confirmatory factor analysis indicated that psychological richness in the Chinese context has a one-dimensional structure. Similarly, the study by Mauro et al. (2025) found that the PR questionnaire demonstrated high validity and reliability in the Italian setting and that a unidimensional factor structure model achieved good fit indices. These studies indicate that they were conducted on a sample of individuals aged 18 and above.

Few studies have examined the development of a PR questionnaire for individuals under 18. This highlights the need to develop tools for measuring PR in this age group, given that PR in childhood, as an indirect psychological construct, is not a subsequent developmental outcome but rather a developmental capacity that can be supported and nurtured early through practical psychological guidance. This foundation contributes to shaping a generation that is more open, flexible, and sophisticated in its interaction with itself and the world, and one that advances the goals of human development. Achieving this requires reliable indicators capable of assessing PR levels in children—an investment in childhood and in the capacities and potential children possess, and a launchpad toward a safe and healthy adolescence (Arnout, 2025).

Studies have demonstrated the effectiveness of using artificial neural networks in developing psychological questionnaires. The study by Dolce et al. (2020) aimed to develop a methodology that integrates interpretive and predictive modeling to construct a new psychometric questionnaire for assessing pathological traits, drawing on concepts from affective neuroscience and phenomenology. The study included a sample of 604 adults attending psychiatric treatment centers, diagnosed using the Italian version of the SCID-5-PD questionnaire according to the DSM-5 criteria. A 260-item questionnaire was used, covering three domains: affective characteristics (157 items), dissociative phenomena (24 items), and pathological traits (79 items). The factor structure was analyzed using principal component exploratory factor analysis, and a multilayer artificial neural network was trained using a resilient backpropagation algorithm to select the items most predictive of diagnosis. The results showed that the use of artificial neural networks enhance the development of more accurate and effective psychological diagnostic tools that combine theoretical explanatory power with predictive accuracy. The study also recommended the use of artificial intelligence algorithms in the development of psychometric tools.

Similarly, Glöckner et al. (2020) developed a novel methodological approach to constructing a comprehensive standard score based on a range of psychological measures using artificial neural networks. They named this standard score the “NeuroNetSuperscore”. The study included 804 participants from Germany who underwent various psychological assessments, including the Big Five personality assessment, mental health measures such as life satisfaction, depression, and anxiety, as well as cognitive performance tests and behavioral indicators. The results demonstrated the superiority of the neural network model in explaining differences related to psychological and cognitive aspects. It also achieved higher predictive performance in predicting mental health-related behaviors and self-reports, reflecting the ability of artificial neural networks to integrate and reduce multidimensional psychological data to a unified index with high clinical and predictive significance. This study recommended the broader application of artificial neural network analysis in psychometrics.

More recently, Wang et al. (2025) investigated the prediction of personality traits and psychological symptoms from behavioral dynamics using machine learning techniques. The study sought to examine the relationships among facial expressions, body movements, and psychological indicators, and to develop effective predictive models based on behavioral characteristics extracted from video clips. The study involved 167 participants from diverse age groups and occupations, who were assessed for psychological symptoms using the SCL-90 Psychological Symptoms Questionnaire and for personality traits using the BFI-2. The results demonstrated the effectiveness of machine learning techniques for non-invasive and accurate assessment of psychological status. In the Arab context, Al-Shaibani (2024) applied neural networks to assess the psychometric properties of the Statistics Anxiety Questionnaire. The study used a sample of 455 students from Taif University who were administered the Vigil-Colet et al. (2008) Statistics Anxiety Questionnaire. The results showed good agreement between the three-dimensional model and the sample data. Furthermore, neural network analysis enabled the extraction of indicators such as “intermediate”, “proximity”, and “intensity”, which helped identify the most influential elements shaping the concept of statistics anxiety, particularly the test-anxiety dimension.

In the same vein, a study by Qasim and Amer (2024) analyzed the structure of the abbreviated version of the Smartphone Addiction Questionnaire developed by Kwon et al. (2013) in the context of Arab society, targeting university students. The study aimed to estimate the prevalence of this type of addiction and examine its associations with quality-of-life factors, using factor analysis and psychometric based- neural network analysis. The study was administered electronically to a sample of 418 male and female students from several Arab countries, who were given the abbreviated version of the Smartphone Addiction Questionnaire, along with a quality-of-life questionnaire. The results of the EFA revealed two main factors that accounted for 64.29% of the variance in addiction scores. The three-factor model (negative impact, dependency, and time spent) showed good fit with the sample data.

The results of the psychometric neural network analysis indicated the importance of several items, such as feeling confused when away from the phone, mental preoccupation with it, and loss of focus due to it. In reviewing the theoretical and research literature, it becomes evident that artificial neural networks (ANNs) have recently gained acceptance and widespread adoption after nearly 50 years of existence. However, given the widespread adoption of powerful computational tools in contemporary society, ANNs have experienced a significant resurgence, greatly benefiting both experts and consumers. Current developments in deep learning and ANNs focus heavily on their ability to model and interpret complex data and on their scalability through optimization and parallelization (Azzolina et al., 2019; Jafarian et al., 2013; Kim, 2016; Li et al., 2021; Zhou et al., 2019; Zappone et al., 2019). Thus, this study aimed to apply artificial neural networks (ANNs) to the factor analysis of the PR questionnaire for children across diverse cultural contexts. The goal was to develop an integrated psychometric model that combines modern statistical techniques with rigorous measurement methods, thereby strengthening the validity of assessment tools and improving their capacity to accurately and objectively detect cross-cultural psychometric variations.

## 2. The Current Study

The concept of PR is a relatively recent dimension in psychological well-being research, introduced by Oishi et al. (2019a, 2019b) as a third explanatory model, alongside happiness and meaning as the two traditional dimensions. This concept has gained increasing scholarly attention in recent years. Despite its development in adult research, interest in children remains limited, if not absent, both theoretically and in the availability of measurement tools and in the study of differences in this variable. Most existing tools focus primarily on traditional indicators of psychological well-being, neglecting aspects of PR that may be crucial in the early stages of development. Children are particularly sensitive to their environment and are most capable of shaping their psychological identity through diverse and profound experiences and enriching interactions (Oishi & Westgate, 2022).

Arnout (2025) stated that PR is a dynamic process that evolves with each stage of development, and that childhood provides fertile ground for the earliest seeds of PR. This is due to the high degree of psychological flexibility, the abundance of opportunities for sensory and experiential learning, and the initial formation of self-narrative. Children raised in environments that foster curiosity, questioning, and experimentation develop flexible and diverse cognitive patterns, characteristics closely associated with PR in early childhood. Furthermore, according to Bowlby’s attachment theory, a child raised in a safe and supportive environment develops confidence and the capacity to form healthy relationships in adulthood. For this reason, Erikson emphasized the importance of building trust, rather than cultivating distrust, at this stage as a foundation for psychological well-being and integration into a richer, deeper life, open to mystery and renewal. Therefore, the need to develop standardized indicators of PR in childhood contexts becomes evident. Such indicators can help identify patterns of experimental restriction or reduced cognitive-emotional stimulation—factors that the literature associates with potential benefit from targeted support. In this sense, these indicators inform the design of individualized interventions, when appropriate, that take cultural, environmental, and social differences into account, thereby enhancing the counselor’s ability to tailor support to each child’s specific developmental needs.

In light of this, the absence of a specific questionnaire for PR in children represents a knowledge and methodological gap that warrants attention, particularly given the growing global focus on promoting children’s psychological well-being as part of broader humanitarian and educational goals. Developing an accurate questionnaire for this group necessitates a deep understanding of their developmental characteristics and their limited ability to articulate their feelings and thoughts. This requires carefully crafting the items and analyzing their structure to ensure validity and reliability.

Furthermore, relying solely on traditional statistical methods—such as confirmatory factor analysis—may be insufficient to capture the complex, nonlinear relationships among questionnaire items, particularly when applied in diverse cultural contexts. This highlights the importance of using artificial neural networks as machine learning tools for detecting complex, nonlinear patterns in data, thereby enhancing model accuracy and deepening the understanding of factor structure across diverse cultural environments (Blei & Smyth, 2017; Casella et al., 2023, Goodfellow et al., 2016; Pokropek & Davidov, 2021). The significance of these efforts extends beyond methodological considerations to encompass practical dimensions related to important cognitive and social factors. Psychological enrichment enhances a child’s psychological resilience, readiness to learn, and ability to respond positively to challenges. These capabilities are essential for achieving the Sustainable Development Goals, particularly Goal 3 on good health and well-being and Goal 4 on inclusive and equitable quality education (Arnout, 2025).

Given the limited research attention to children’s PR in the Arab world and the absence of tools to measure it, this study aimed to develop a PR questionnaire for children that is both methodologically rigorous and culturally appropriate. The current study sought to answer the following questions:

**RQ1.**  *What is the latent factor structure of the children’s PR questionnaire?*

**RQ2.**  *What is the optimal factor structure for the children’s PR questionnaire?*

**RQ3.**  *Which measurement indicators have the most significant influence on the structure of the concept of children’s PR?*

**RQ4.**  *Does the children’s PR questionnaire possess good reliability indicators?*

**RQ5.**  *Are there differences in PR between children in the Saudi and Egyptian environments?*

## 3. Methods and Materials

This study adopted a descriptive approach to develop a psychological richness questionnaire for children (prepared by the researcher). The study targeted children residing in the Kingdom of Saudi Arabia and the Arab Republic of Egypt who were under 18 years of age. The researcher selected a stratified random sample of 1041 children, aged between 10 and 17 years, with a mean age of 12.94 ± 2.14. This sample included 440 children from the Kingdom of Saudi Arabia, with a mean age and standard deviation of 12.91 ± 2.15, and 601 children from the Arab Republic of Egypt, with a mean age and standard deviation of 12.97 ± 2.14. Written informed consent was obtained from the parents of the participants. Ethical approval was obtained from the Institutional Review Board (IRB) of the Welfare Children’s Association (IRB Log Number: 27437). Responses from participants over the age of 17, according to the legal definition of a child, were excluded. Table 1 presents the demographic characteristics of the study sample.

### 3.1. Materials

#### Children’s PR Questionnaire

The researcher conducted a comprehensive review of PR questionnaires in general and, specifically, those for children. This review led the researcher to adopt the Oishi et al. (2019a) questionnaire, which, to the researcher’s knowledge, is the only questionnaire currently available, due to the novelty of the concept. It has been translated into several languages, and its factor structure and validity across different cultures have been well-established. Studies on PR were also reviewed, such as Liu et al. (2024); Mauro et al. (2025); Oishi and Westgate (2025); and Oishi et al. (2019a, 2019b, 2020, 2025). In light of the absence of measures for children’s PR, the researcher developed a new PR questionnaire for children aged 10 to 17. This self-reported questionnaire was designed to assess key indicators of PR. The initial version consisted of 25 items and was reviewed by 10 experts in psychology and psychometrics. The review process led to the removal of five items, yielding a final 20-item questionnaire. A revised version was then developed for the study sample after the items were edited and linguistically corrected. The researcher selected a five-point Likert scale for each item, with response options of 1 to 5.

### 3.2. Data Analysis

To assess the factor structure of the researcher-developed children’s PR questionnaire, exploratory factor analysis (EFA) was conducted using principal component analysis (Jolliffe, 2002), with the criterion for item loadings set at 0.40 (Hair et al., 2010). To determine the optimal factor structure for the children’s PR questionnaire, confirmatory factor analysis was conducted using the Akaike information criterion (AIC) and Bayesian information criterion (BIC). A psychometric analysis of an artificial neural network was also applied to identify the most influential indicators of the PR concept structure in children. This was conducted using the EBICglasso method to compute the weight matrix for the questionnaire items and to obtain centrality plot measures (structure, convergence, strength, and influence). Reliability was assessed using Cronbach’s α and McDonald’s ω. To detect differences between children in the Saudi and Egyptian environments, a Bayesian independent-samples *t*-test was applied. All statistical analyses were performed using JASP 0.19.2.0 software.

## 4. Results

The results of RQ1, which asked: What is the latent factor structure of the children’s PR questionnaire? To answer this question, the researcher investigated the factor structure of the children’s PR questionnaire in the total study sample and in the Saudi and Egyptian samples using EFA with principal component analysis (PCA). The EFA results for the total sample (1041) showed that the value of the Kaiser–Meyer–Olkin (KMO) criterion was 0.983, indicating a highly adequate sample size for factor analysis. The Bartlett test (χ^2^ = 27,701.494) and degrees of freedom (190, <0.001) also indicated the adequacy of the analysis sample. The EFA also yielded a single factor, “psychological richness”, with a latent root of 15.277. All items on the questionnaire were saturated with this factor, which explained 76.40% of the total variance in children’s PR. This indicates the effectiveness of all 20 questionnaire items in assessing the PR structure within the study sample. Furthermore, the communalities values for all items on the questionnaire exceeded 0.50, indicating the absence of measurement error in the data collected from the study sample.

The results of the EFA, presented in Table 2, indicate high factor loadings for the questionnaire items, with values ranging from 0.734 to 0.919. This demonstrates a strong correlation between each item and the assumed overall factor. These high values reinforce the internal consistency of items within a single dimension and support the validity of the questionnaire’s one-dimensional factor structure in the total sample. It is noteworthy that all items exceeded the minimum acceptable factor loading threshold (0.40) according to Costello and Osborne’s (2005) criteria, with the vast majority approaching or exceeding (0.85). This is considered a strong indicator of clear differentiation within a one-dimensional factor, as noted by Field (2018). In particular, item 1 showed the highest loading (0.919), followed by item 20 (0.917), highlighting their central role in representing the overall concept of PR in children.

In the same context, the EFA analysis for the Saudi sample (n = 440) yielded a KMO value of 0.981, indicating a highly adequate sample size for factor analysis. The Bartlett test value was (χ^2^ = 12,644.862) with degrees of freedom (190, <0.001), and the chi-square value was (1139.960) with degrees of freedom (170, <0.001) confirming the suitability of the data for factor extraction. The EFA also yielded a single-factor, “PR”, with a latent root of 15.928. This factor accounted for 79.60% of the total variance in PR among Saudi children, indicating the effectiveness of all questionnaire items in measuring the PR structure of the sample. The results also showed that the communality values for all items on the questionnaire exceeded 0.50, indicating that the data collected from the Saudi sample were free from substantial measurement error.

The results in Table 3 on the factor saturation of the questionnaire items indicate that all items of the PR questionnaire for the sample of children in Saudi Arabia showed high loadings on the overall factor in the EFA, with values ranging from 0.853 to 0.924. These results reflect a strong correlation between the items and the assumed underlying factor that measures PR. Loadings exceeding 0.70 indicate high psychometric quality, whereas values approaching or exceeding 0.90, as in the first and twenty items, indicate a strong and accurate conceptual representation of the questionnaire’s theoretical content, according to Field’s (2018) criterion.

These results support the one-dimensional factor structure hypothesis, indicating that all items are related to a common general factor and that no additional subfactors are required. From a psychometric perspective, the high and closely spaced loadings indicate strong construct validity and enhance confidence in using the total score as an accurate scientific indicator of PR (Costello & Osborne, 2005). Thus, the data confirm that the children’s PR questionnaire possesses robust psychometric properties that support its adoption in psychological and educational research targeting children in the Saudi community, while ensuring internal consistency and adequate representation of the underlying factor structure (Fabrigar et al., 1999).

This one-dimensional structure simplifies interpretation and application, as the total score can serve as a clear indicator of PR level without subdividing it into multiple, disparate sub-dimensions. This structure also demonstrates that the items were designed or selected to ensure functional and conceptual coherence around a common psychological factor, indicating high internal consistency and aligning with the high factor loadings identified in the previous analysis.

Additionally, the EFA analysis of the Egyptian sample (n = 601) yielded a KMO criterion value of 0.982, indicating strong intercorrelations among items and suitability for factor analysis. The Bartlett test value was 15,289.822 (χ^2^ = 190, <0.001), and the chi-square (χ^2^ = 673.870, 151, <0.001) confirmed the adequacy of the sample for factor extraction sample. The EFA also yielded two factors; the first, “diversity of experiences”, had a latent root value of 14.859, and items 1–11 loaded onto it. This factor accounted for 74.30% of the variance in the correlation matrix among the items loaded onto it in the Egyptian sample. The second factor, “deep knowledge and intellectual openness”, was loaded onto nine items, with a latent root mean square (RMSE) of 1.022; this factor explained 5.10% of the variance in the correlation matrix for items 12–20, which is lower than that of the first factor. The factor extraction was conducted using an oblique rotation method, specifically Promax rotation. The results also showed that the two factors (diversity of experiences and deep knowledge and intellectual openness) together accounted for 79.40% of the total variance in the correlation matrix among the questionnaire items as a whole. This indicates the effectiveness of all questionnaire items in measuring the PR structure of the sample of children from the Arab Republic of Egypt. The results also showed that the communality values for all items on the questionnaire exceeded 0.50, indicating that the data collected from the research sample were free of measurement errors.

The results of the EFA presented in Table 4 indicate that the PR questionnaire for children in the Egyptian context comprises two main factors. The latent root of the first factor reached 14.859, explaining 74.30% of the total variance, while the latent root of the second factor reached 1.022 and explained a limited percentage of 5.10%. This indicates the dominance of the first factor (diversity of experiences) and reduced the relative influence of the effect of the second factor (cognitive depth and intellectual openness). This indicates that the most important factor in PR is the experiential aspect, whereas the cognitive aspect is a consequence of experiential diversity. This leads to treating the questionnaire structure as close to univariate despite the extraction of two factors. In terms of item loads, items (1–11) were strongly correlated with the first factor, with loads ranging from 0.705 to 0.883, all of which exceeded the acceptable limit of 0.40, and most of them even exceeded 0.75, reflecting high structural consistency and an indication of a unified psychological dimension measurement. Items (12–20) were also strongly correlated with the second factor, with loadings ranging from 0.714 to 0.890; however, the limited variance explained by the second factor reduces the importance of the binary model and makes reliance on it weak unless a clear theoretical justification for dividing the items is provided. Based on these indicators, the two-factor model appears to enhance the questionnaire’s validity and suggests a well-consistent factorial structure. However, the significant difference in the interpretation of the variance between the two factors strongly supports the one-factor hypothesis. This opens the door to interpreting the factorial structure as either a dominant, one-dimensional model or a two-dimensional, interrelated questionnaire with one primary and one secondary dimension.

The factor structure indicates that the items represent two interrelated dimensions within the questionnaire framework, such that RC1 and RC2 each account for distinct patterns of children’s responses. This dual structure supports the use of sub-scores alongside the overall score, particularly when item segmentation is based on a clear theoretical foundation. In the same vein, factor RC1 exhibits greater coherence and consistency, as evidenced by stronger correlations with its items. This aligns with the quantitative analysis results, which confirmed its superiority in explaining variance compared to RC2. This suggests the possibility of developing a short version of the questionnaire consisting of 11 items (1–11), as it best represents the concept of PR among children in the Egyptian context.

For RQ2, which asked: What is the optimal factor structure for the children’s PR questionnaire? to answer this question, the researcher conducted confirmatory factor analysis (CFA) to determine the optimal factor structure for the children’s PR questionnaire across the total study sample and the Saudi and Egyptian samples, and computed fit indices. The following are the research findings (Table 5):

The results in Table 5 indicate that the two-factor model (diversity of experiences, depth of knowledge, and intellectual openness) outperformed the one-dimensional factor model across all three samples (Saudi, Egyptian, and total). This superiority is evident from the lower chi-squared value in the two-factor model compared to the one-dimensional-factor model.

The following presents the loadings and their significance for the children’s PR questionnaire across the three samples (Saudi, Egyptian, and total).

The results in Table 6 for the CFA of the Saudi Arabian factor model indicate a strong relationship between the items and the two assumed factors in the children’s PR questionnaire. The item loadings for the first factor were high, ranging from 0.808 to 0.978, demonstrating good homogeneity among these items and their apparent ability to represent this factor. Furthermore, all of these loads fell within the 95% confidence interval and were relatively stable, thereby enhancing the reliability of the estimates. In contrast, the items associated with the second factor generally showed even higher loadings, ranging from 0.995 to 1.144, with a notably lower standard error, reflecting a high degree of reliability in the estimates and accuracy in the measurement. According to Brown (2015), factor loadings exceeding 1.0—known as Heywood cases—are not necessarily problematic. In fact, they may indicate high explanatory power in specific cases, provided that other indicators within the model support these estimates (Lima-Castro et al., 2021).

In the Egyptian context, the confirmatory factor analysis results, as shown in Table 7, were consistent with those obtained in the Saudi context, with minor variations. The loadings of the items on the first factor ranged from 0.791 to 0.956, indicating a strong structural correlation between these items and the factor, notably since all exceeded the minimum acceptable threshold of 0.70 (Kline, 2016). As for the second factor, the loadings ranged from 0.940 to 1.115, and these estimates were supported by relatively narrow confidence intervals, thus reinforcing the reliability of the PR questionnaire in the Egyptian context. It is noteworthy that the high, similar saturation values for this factor indicate good internal homogeneity, which in turn supports the structural validity of this factor within the overall questionnaire structure.

Furthermore, the results of the total sample, as shown in Table 8, enhance the reliability of the two-factor model across different environments. The loadings of the items associated with the first factor ranged from 0.814 to 0.967, with very low standard errors of 0.018 for most items, confirming the model’s accuracy and the reliability of its results. Similarly, the loadings of the items associated with the second factor were high (0.975–1.126), indicating a strong relationship between these items and the second factor and supporting the theoretical hypothesis of a distinct psychological dimension for this group of items. It was also observed that the consistency of confidence intervals at both the lower and upper limits, as well as the recurrence of saturation patterns across different environments, reflects a high degree of homogeneity in the questionnaire structure and enhances its generalizability and applicability across multiple cultural contexts. This aligns with the recommendations of studies that have addressed cross-cultural validity assessment, such as those by G. W. Cheung and Rensvold (2002), and also confirms the findings of previous research and studies on the validity of the PR questionnaire in diverse cultures (Arnout, 2025; Liu et al., 2024; Mask et al., 2025; Mauro et al., 2025; Oishi et al., 2019a; Oishi & Westgate, 2022). Accordingly, the results presented in Table 6, Table 7 and Table 8 reflect the strength and robustness of the questionnaire’s factor structure across the Saudi, Egyptian, and overall samples. The high loading values, low standard errors, and stable confidence intervals indicate strong adherence to the proposed questionnaire. These results support the use of the binary model as a basis for assessing children’s PR across cultural contexts and underscore the importance of developing measurement tools grounded in sound normative foundations that enable accurate and objective observation of individual and cultural differences.

**Figure 1:**
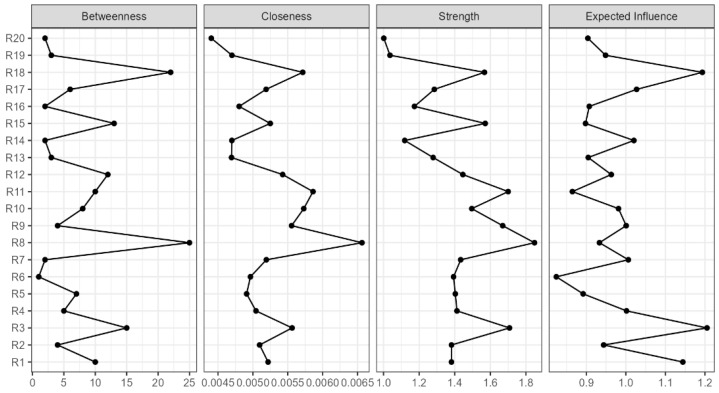
Graphical representation of the centrality scheme for items of the PR questionnaire for children in the Saudi environment.

**Figure 2:**
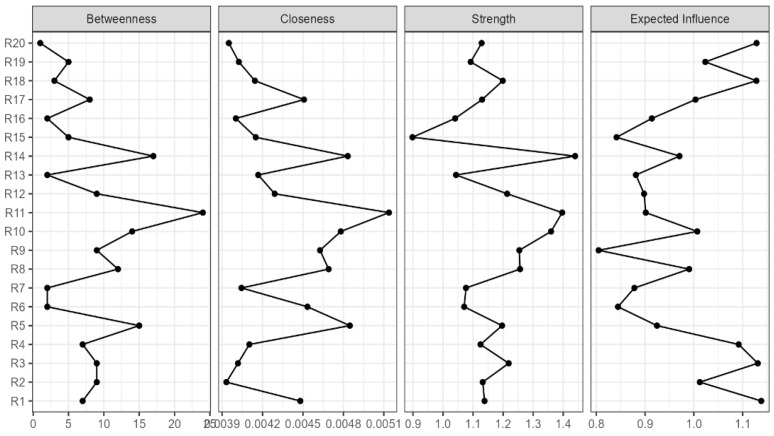
Graphical representation of the centrality scheme for items of the PR questionnaire for children in the Egyptian environment.

**Figure 3:**
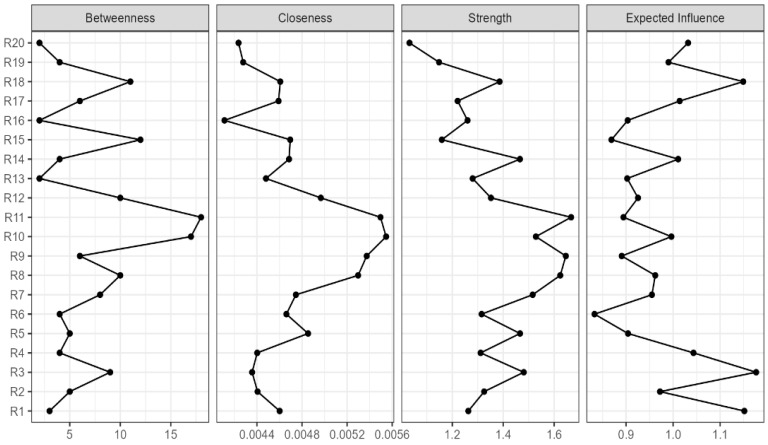
Graphical representation of the centrality scheme for items on the children’s PR questionnaire in the total sample.

**Figure 4:**
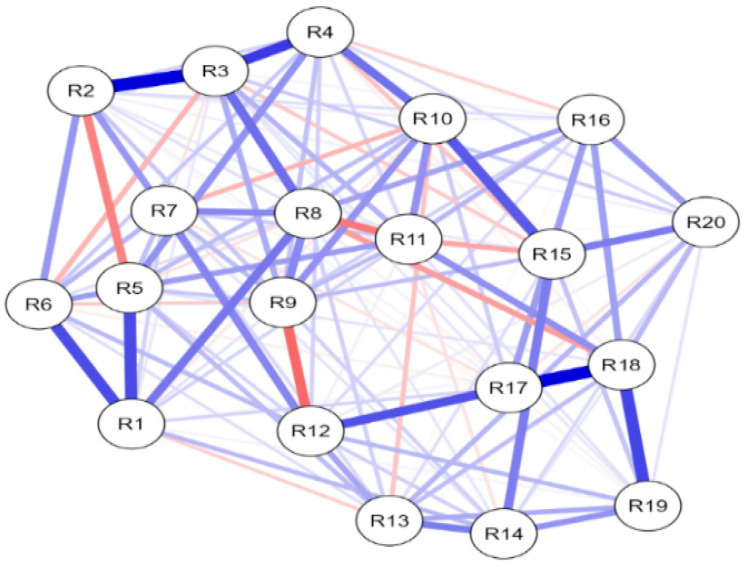
Psychometric neural network for the PR questionnaire for children in the Saudi environment.

**Figure 5:**
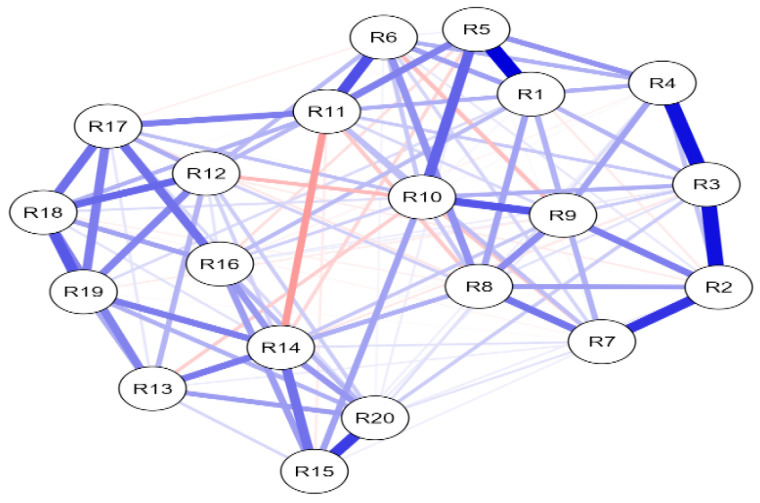
Psychometric neural network for the PR questionnaire for children in the Egyptian environment.

**Figure 6:**
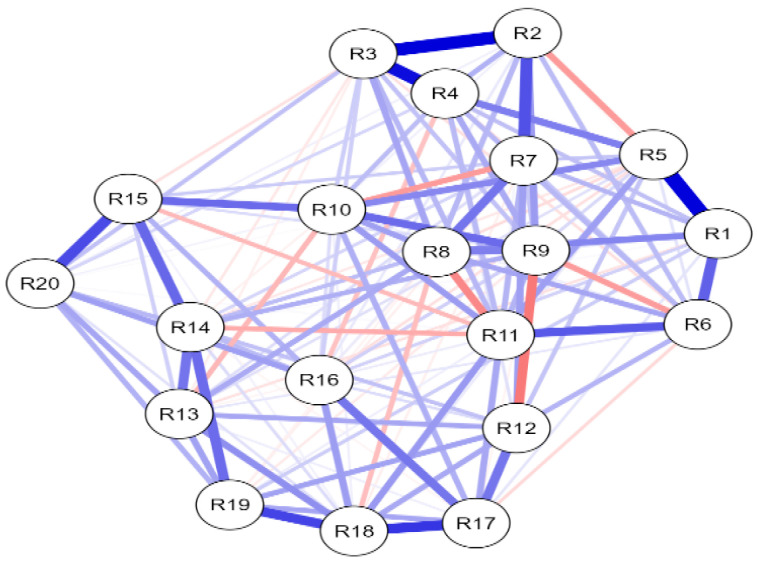
Psychometric neural network for the PR questionnaire for children in the total sample.

To answer RQ3, which stated: Which measurement indicators have the most significant influence on the structure of the concept of children’s PR? the researcher conducted a centrality schema analysis using psychometric neural network analysis, specifically the EBICglasso estimation method. The psychometric neural network analysis of the PR questionnaire in the three samples yielded the following results regarding the centrality diagram measurements of the importance of the items and the contribution of each item to the formation of the questionnaire’s neural network (Table 9, Table 10 and Table 11 and Figure 1, Figure 2, Figure 3, Figure 4, Figure 5 and Figure 6):

The results presented in Table 9, Table 10 and Table 11 indicate that the analysis of the centrality scheme of the items on the children’s PR questionnaire, using a psychometric neural network, revealed multiple significances based on betweenness, closeness, strength, and potential influence indices. This reflects apparent variation in the importance of the items and their structural contributions across the Saudi, Egyptian, and total samples.

To answer RQ4, Does the Children’s PR Questionnaire possess good reliability indicators? the researcher analyzed the questionnaire’s reliability indicators in the Saudi, Egyptian, and total samples using Cronbach’s α and McDonald’s ω. Table 12 presents the results.

The results presented in Table 12, regarding reliability testing using Cronbach’s alpha and McDonald’s omega, indicate a high level of reliability for the children’s PR questionnaire across all three samples (Saudi, Egyptian, and total) at both the sub-factor and questionnaire levels. In the Saudi sample, Cronbach’s α was 0.978 for the first factor, 0.975 for the second, and 0.987 for the questionnaire as a whole. McDonald’s ω reliability indices were very similar: the first factor scored 0.979, the second 0.975, and the overall reliability of the questionnaire was 0.988, indicating strong reliability across the instrument’s components in this sample, as noted by Field (2018).

Similarly, the Egyptian sample showed comparable results for the questionnaire’s reliability coefficient: Cronbach’s α was 0.967 for the first factor, 0.972 for the second, and 0.981 for the questionnaire as a whole. Similarly, the reliability results for the MacDonald ω were comparable across the two samples: 0.968 for the first factor, 0.972 for the second factor, and 0.983 for the questionnaire as a whole, indicating a high degree of structural agreement between the Saudi and Egyptian samples. Overall, the indicators showed high reliability: Cronbach’s α was 0.972 for the first factor, 0.973 for the second, and 0.983 for the overall questionnaire. The MacDonald ω reliability values were similar, with 0.973 for both factors and 0.985 for the questionnaire as a whole, confirming the instrument’s reliability across diverse contexts.

**Figure 7:**
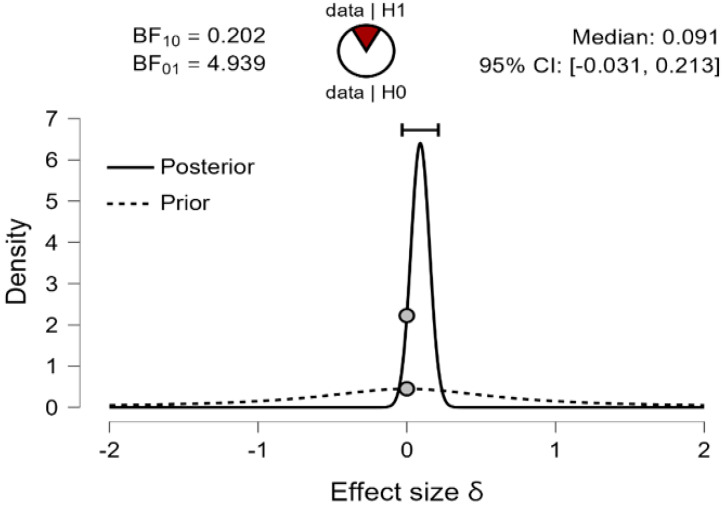
Bayesian probability density scheme for effect size.

**Figure 8:**
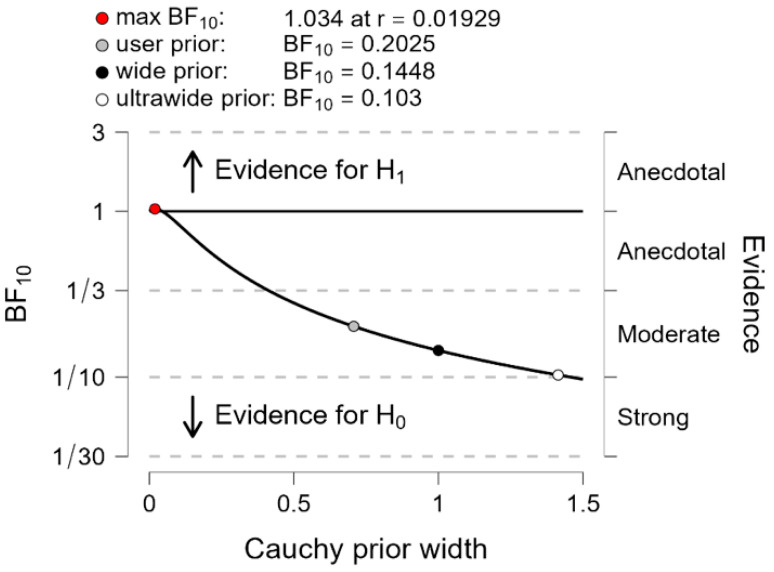
Bayesian factor robustness check for differences in PR between children in the Saudi and Egyptian environments.

**Figure 9:**
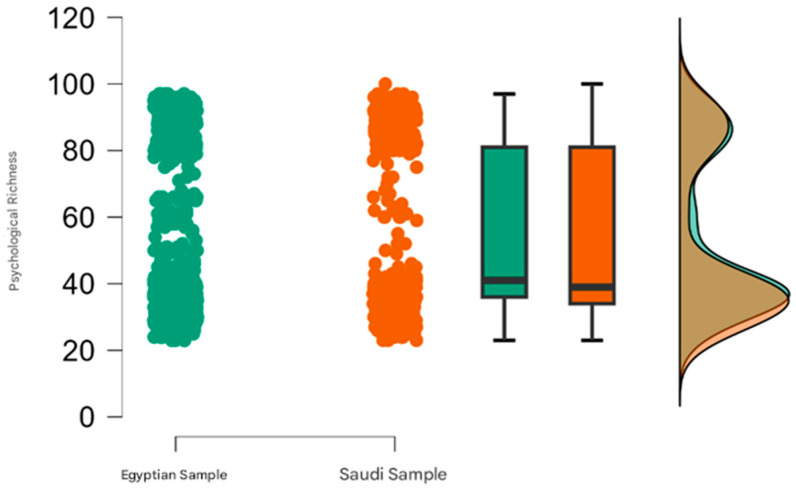
Descriptive statistical density cloud for differences between the Saudi and Egyptian samples in PR.

Regarding RQ5, are there differences in PR between children in the Saudi and Egyptian environments? the researcher used a Bayesian independent-samples *t*-test to assess differences and their significance in PR between Saudi and Egyptian research participants. Table 13 shows the results:

The results shown in Table 13 indicate that the strength of the Bayesian Facility Index (BF10) for the differences in PR between children in the Saudi and Egyptian environments was very weak (0.202), according to the Jeffrey criterion (weak if the Bayesian Facility value is between 1 and 3). This supports the null hypothesis, which indicates that there are no differences in PR between the children in the Saudi and Egyptian environments within the research sample. The mean scores for the Saudi sample in PR were M = 51.97 (χ^2^ = 24.649), whereas those for the Egyptian sample were M = 54.18 (χ^2^ = 23.545). Therefore, the alternative hypothesis was rejected in light of these results, as shown in Figure 7, Figure 8 and Figure 9.

## 5. Discussion

Factor analysis of the questionnaire revealed a unidimensional structure for both the overall sample and the sample of children in the Saudi context, in contrast to a two-dimensional structure in the Egyptian context, although the first factor accounted for most of the variance. This makes the model closer to a unidimensional model. These results are consistent with the hypothesis of Oishi et al. (2019b) that PR has a unidimensional factor structure. This finding also aligns with the conclusions of numerous studies on the factor structure of the PR questionnaire developed by Oishi et al. (2019a), whose results confirmed the single-factor structure (among these studies are Arnout, 2025; Liu et al., 2024; Mask et al., 2025; Mauro et al., 2025; Oishi et al., 2020; Oishi & Westgate, 2022).

Additionally, the findings on the optimal factor structure for the children’s PR questionnaire indicated that the two-factor model (diversity of experiences, depth of knowledge, and intellectual openness) was superior to the one-dimensional factor model across all three samples (Saudi, Egyptian, and total). For example, the chi-squared value for the two-factor model in the Saudi sample was 717.880 compared with 1152.893 for the single-factor model, indicating a significant improvement in the model’s fit to the data. When considering the comparative fit indices, namely CFI and TLI, the results were even more positive, favoring the two-factor model across all three samples. These indices exceeded the accepted reference value of 0.95 in most cases, as noted by Bentler (1990), indicating a high quality of fit. For example, the CFI and TLI values in the Egyptian sample were 0.968 and 0.964, respectively, in the two-factor model. In contrast, their values in the general factor model did not exceed 0.894 and 0.881, demonstrating a clear advantage of the two-dimensional structure over the general factor model.

Similarly, the RMSEA results support this trend; its value in the two-factor model fell below 0.08, which is the threshold established by Browne and Cudeck (1993) and G. W. Cheung and Rensvold (2002) and is considered the acceptable limit for indicating good fit. For example, in the overall sample, the RMSEA value decreased from 0.120 in the general factor model to 0.075 in the two-factor model, reflecting a significant improvement in the structural fit of the measure. Furthermore, the relative efficiency indices, namely AIC and BIC, showed a significant decrease for the two-factor model relative to the general factor model. This indicates greater efficiency in balancing fit quality and model complexity, as explained by Burnham and Anderson (2004). For example, in the Egyptian sample, the two-factor model yielded an AIC of 26,847.432, compared with 27,978.756 for the general factor model, thereby reinforcing the preference for the simpler model without compromising representativeness. Based on the findings, the two-factor model provides a more accurate and appropriate representation of the questionnaire’s assumed structure across samples. The goodness-of-fit indices clearly demonstrate that the two-dimensional structure is more effective at explaining the interrelationships among items, supporting the instrument’s structural validity and confirming the necessity of adopting the model that best fits the data from both statistical and conceptual perspectives. Therefore, the two-factor model was adopted for the children’s PR questionnaire in both the Saudi and Egyptian contexts.

Furthermore, the psychometric neural network analysis of the PR questionnaire in the Saudi, Egyptian, and total sample results regarding betweenness showed that items 8, 18, 3, 15, 12, 11, and 10 were the most mediated in terms of relationships between any two nodes or items in the neural network of the children’s PR questionnaire in the Saudi context, while in the Egyptian context, items 11, 14, 5, 10, and 8 were the most mediated. In the total sample, items 11, 10, 15, 18, 12, and 8 were the most mediated. Furthermore, the results regarding closeness showed that its value in the Saudi sample ranged from 0.004 to 0.007, with item 20 being closest to the other items on the questionnaire.

While in the Egyptian sample the closeness values ranged between 0.004–0.005 and items 1, 12, 13, 15, 16, and 18 were the closest of the other items in the neural network of the questionnaire, in the total sample, the closeness values ranged between 0.004–0.006, and items 13, 16, 19, 2, and 20 were the closest. In addition, the results of the strength index in the Saudi sample showed that items 16, 3, 11, 9, 15, 18, 10, 12, and 7 were the strongest and most connected in the neural network of the questionnaire. In contrast, in the Egyptian sample, the results showed that the strongest items were 14, 11, 10, 8, 9, 3, 12, and 18, while in the total sample, the strongest items were 11, 9, 8, 10, 7, 3, 5, and 14. The results of the expected influence criterion also showed that items 3, 18, 1, 17, 14, 7, and 4 were likely to have the most significant influence compared to the other items on the questionnaire in the Saudi sample.

Also, in the Egyptian context, items 1, 3, 20, 18, 4, 19, 10, and 17 had the most substantial expected influence on measuring children’s PR compared with the other items. However, in the total sample, items 3, 1, 18, 4, 20, 17, and 14 were likely to have the most significant influence compared to the other items on the structure of the concept of children’s PR.

In the Saudi context, item R8 stood out as the most central item in terms of interrelationship (25.000), convergence (0.007), and power (1.848), indicating its vital role in linking the questionnaire’s components and guiding the flow of information within the network. This finding aligns with previous studies that confirmed that high-interface elements act as bridges connecting different conceptual clusters (Opsahl et al., 2010). Other items, such as R3, R18, and R11, also demonstrated significant levels of strength and expected influence, reflecting their clear contribution to organizing relationships within the questionnaire and their effectiveness as psychological indicators for interpreting the conceptual structure of children’s PR.

In the Egyptian context, item R11 ranked highest on the interface index with a score of 24,000, despite its low expected impact of 0.901. This may indicate that its role is structural in linking without a significant psychological or emotional impact. Neural network research addresses this paradox, confirming that structural centrality does not always correspond to the expected psychological effect (Bringmann et al., 2019). Conversely, items R3, R18, and R20 showed more substantial expected influence, indicating their effectiveness in eliciting children’s perceptions of psychological fulfillment and well-being and their role in shaping the meaning and emotion networks associated with the concept of PR.

Across the total sample, items R3, R8, and R9 had high strength and correlation coefficients of 1.481, 1.624, and 1.646, respectively, indicating their consistent centrality within the network structure even after combining the Saudi and Egyptian samples. This finding supports the theoretical literature’s assertion that pivotal elements retain their relative importance when the analysis is broadened, thereby enhancing the questionnaire’s explanatory power and diagnostic flexibility across cultures (Epskamp et al., 2018). Item R10 also recorded the highest interstitial value (17,000) despite its modest psychological impact, indicating its role as a conceptual mediator between disparate components—a fundamental characteristic for understanding the internal structure of psychological questionnaires.

Furthermore, Figure 4, Figure 5 and Figure 6, each depicting a network of relationships among 20 labeled nodes (R1 through R20), indicate a clear degree of homogeneity in the interactions of items with the questionnaire dimensions across the Saudi and Egyptian contexts, as well as in the overall sample. In Figure 4, which pertains to the Saudi context, the items are concentrated within a clear semantic range centered on the factors, highlighting their strong correlation with the main factors. This concentration reflects cognitive and behavioral consistency in Saudi children’s responses and the questionnaire’s ability to capture the theoretical concept of PR with high accuracy. Figure 5 also illustrates a symmetric distribution in the Egyptian sample, indicating partial cultural convergence, which enhances the validity of the children’s PR questionnaire for measurement across Arab contexts. Similarly, Figure 6, representing the overall research sample, highlights greater coherence in the distribution of items around the central axes, indicating the integration and complementarity of responses and supporting the questionnaire’s structural stability.

Accordingly, the centrality diagrams for the Saudi, Egyptian, and overall research samples provide strong evidence of the questionnaire’s internal construct validity and demonstrate its effectiveness in representing the concept of PR in children within similar Arab cultural environments. These results also indicate the integration of centrality indicators in forming an accurate structural map that reveals the contribution of each item within the questionnaire’s framework. This will pave the way for further development of the researcher’s PR questionnaire for children and its adaptation to specific cultural and contextual characteristics. These findings are consistent with current trends in artificial neural network analysis for psychological research (Costantini et al., 2015). These results are consistent with previous research and studies on the effectiveness of using artificial neural networks and centrality mapping analysis in identifying the most influential indicators in the structure of the concept to be measured (Al-Shaibani, 2024; Qasim & Amer, 2024; Dolce et al., 2020; Milad et al., 2021; Mosavi et al., 2019; Nabipour et al., 2020; Oliver et al., 2021; Šahović et al., 2012; Wang et al., 2025; Zhou et al., 2019).

Furthermore, the results of the current study, presented in Table 12, for reliability testing using Cronbach’s alpha and McDonald’s omega methods, indicate a high level of reliability for the children’s PR questionnaire in all three samples (Saudi, Egyptian, and the total sample), both at the sub-factor and questionnaire as a whole.

Additionally, the results showed no differences in PR between children in the Saudi and Egyptian environments. Figure 9 shows the Bayesian probability density plot for the effect size of the results of the Bayesian independent samples *t*-test to study the differences between the Saudi and Egyptian samples of the research sample in PR, where the average scores of the Saudi sample (51.97) did not differ much from the average scores of the Egyptian sample (54.180) in PR; the Bayesian coefficient for the alternative hypothesis H_1_ was very low (BF_10_ = 0.202) compared to the Bayesian coefficient for the null hypothesis H_0_ (BF_01_ = 4.939). This means that the data support the null hypothesis approximately five times as strongly as the alternative hypothesis. The tiny median effect size further supports this (Cohen’s δ = 0.091, 95% CL = −0.031–0.213), and the confidence interval was zero, which reinforces the absence of statistically significant differences in PR between children in the Saudi and Egyptian environments.

As shown in Figure 7, the strength of the Bayesian factor test for differences between the Saudi and Egyptian samples (BF_10_) reached a maximum of 1.034 for PR when the data prior distribution was set to a very narrow width (0.01929). With increasing prior distribution width, the value of the Bayesian factor (BF_10_) decreased to 0.2025 with the researcher’s data prior, to 0.1448 with a wide prior distribution, and finally to 0.103 with an ultrawide prior distribution. This continuous decrease indicates that the evidence gradually shifts in favor of the null hypothesis (H_0_) as the prior distribution widens. From these results, we conclude that the data are consistent with the null hypothesis, indicating no difference in PR between the Saudi and Egyptian samples of children. In contrast, the alternative hypothesis is supported across a wide range of prior distributions (Figure 7).

Figure 8 shows a remarkable similarity in the distribution pattern of PR scores between children in the Saudi sample and the Egyptian sample in terms of the general shape of the distribution, the median, and the range of values, which is consistent with the previous statistical results that supported the null hypothesis in terms of the absence of substantial differences between children in the Saudi and Egyptian environment in PR. These results highlight the importance of researchers using Bayesian analysis to study differences between samples, given its ability to directly estimate the probability of competing hypotheses across prior and subsequent distributions, rather than relying on traditional statistical significance indicators. This enhances the strength of inference and the accuracy of interpreting results (Arnout, 2024; Du et al., 2020; Kruschke, 2015).

These findings are consistent with those of F. M. Cheung et al. (2011), who found that many psychological indicators, such as psychological well-being and cognitive and emotional functions in childhood, are often characterized by cross-cultural stability. Differences in core psychological traits were not statistically significant despite apparent cultural variations between American and Chinese cultures. They also align with Ryan and Deci’s (2001) assertion that a sense of meaning, appreciation, and belonging stems from universal human needs. Mesquita (2010) indicated that cultural differences may influence patterns of emotional expression and thinking styles, but do not affect the core psychological characteristics of positive traits, especially in children. Childhood is a crucial stage in this process. This is the stage at which the seeds of personality are sown, and they develop throughout subsequent stages of growth (Yarkoni & Westfall, 2017). Therefore, children at this stage are still learning and gaining experiences compared to adolescents and adults. As Oishi et al. (2020) indicated, psychological enrichment is a continuous, dynamic process that grows with the accumulation of experiences throughout a person’s life cycle.

Furthermore, these findings align with the global focus on childhood and on investment in it to achieve the Sustainable Development Goals, as well as with countries’ efforts in this regard. In the Kingdom of Saudi Arabia, Vision 2030 provides a comprehensive strategic framework that includes programs to enhance quality of life by providing family, educational, and health environments that promote mental well-being, and by offering extracurricular activities that develop personality, identity, and belonging. The Ministry of Education has played a prominent role in this by integrating concepts of psychological well-being and societal values into the curriculum, thereby increasing awareness of the importance of cultivating a generation with balanced and positive mental health. Similarly, the Arab Republic of Egypt has made pioneering efforts in child care through its Vision 2030, which emphasizes the social dimension and children’s rights as well as the provision of quality education, healthcare, mental health support, and family and school protection. Numerous programs and initiatives aimed at promoting positive socialization and psychological empowerment among children have been implemented, resulting in improved psychological and social well-being in recent years. Despite cultural differences across countries, there are commonalities in visions and development plans, regardless of geographical background, as well as in viewing childhood as a central pillar of social and economic transformation.

## 6. Conclusions

This research attempted to utilize artificial neural networks to develop a PR questionnaire for children (prepared by the researcher), reveal the underlying factor structure of the questionnaire in the Saudi and Egyptian contexts, identify the optimal factor structure for this questionnaire, determine the most influential standard indicators in the conceptual framework of PR in children, verify the questionnaire’s reliability, and uncover differences in PR between children in the Saudi and Egyptian contexts. The research sample consisted of 1041 children aged 10–17 years. The results of the EFA indicated that the children’s PR questionnaire developed in this study had a one-dimensional factor structure (PR) in both the Saudi and total samples. In contrast, in the Egyptian sample, the EFA resulted in two factors, namely the diversity of experiences or expertise, and the depth of knowledge and intellectual openness, while the results of the confirmatory factor analysis revealed that the two-factor model of the questionnaire had good matching indicators with the data in the three samples, Saudi, Egyptian and the total sample, which indicates the validity of the two-factor model of the children’s PR questionnaire across different cultures. The results of the artificial neural network analysis of the centrality schema indices (structure, convergence, strength, and influence) revealed the significance of questionnaire items 3, 18, 1, 17, 14, 7, and 4 for the Saudi sample, while for the Egyptian sample, items 1, 3, 20, 18, 4, 19, 10, and 17 were significant. In the overall sample, items 3, 1, 18, 4, 20, 17, and 14 were found to be the most influential on the structure of the concept of children’s PR. Furthermore, reliability analyses of the Saudi, Egyptian, and overall samples demonstrated that the children’s PR questionnaire exhibited good reliability across cultural environments. Additionally, the results revealed no significant differences in PR between children in the Saudi and Egyptian environments. In light of the research findings, the study recommends applying neural network analysis to the development and evaluation of psychometric tools, and using the children’s PR questionnaire in psychometric assessment across cultural contexts in the Arab world, given its credibility and the reliability of its factor structure.

### 6.1. Limitations and Future Directions

This study developed a new children’s PR questionnaire, created by the researcher using artificial neural networks, and verified its factor structure in the Saudi and Egyptian contexts. The research yielded highly significant results in assessing children’s PR across diverse cultures. The results showed that the two-factor model was the most suitable, with higher concordance indices, particularly under the Akaike information criterion (AIC) and Bayesian information criterion (BIC), than the one-factor model in both cultural contexts. On the other hand, the analysis identified specific indicators that were most influential within the conceptual framework of PR in the Saudi and Egyptian contexts. This opens the door to diverse future psychometric applications of this concept across multicultural samples, thereby enhancing the possibility of generalizing the questionnaire’s use and adapting it to local contexts. The results also revealed no significant differences between children in the Saudi and Egyptian environments.

Despite the importance of these findings, the research employed only a descriptive approach to verify the questionnaire’s psychometric properties, without including intervention designs aimed at developing PR in children or predictive studies based on relevant variables. Furthermore, the study was confined to the Saudi and Egyptian contexts only and did not extend to other cultural contexts. Additionally, it relied exclusively on self-report measures, without employing alternative methods such as interviews or direct observation. Based on the above, there is a need for future research on PR in children to adopt more diverse research methodologies. The methodologies include quantitative, qualitative, and mixed approaches, as well as the verification of the questionnaire’s factor structure and psychometric properties across different cultural and social contexts. Predictive studies linking PR to demographic and familial variables are also recommended, as well as using diverse measurement tools to enrich the research findings and enhance their objectivity.

### 6.2. Implications for Practice

The PR questionnaire for children, developed in this research, is helpful in educational and counseling contexts for psychometric and screening purposes and has demonstrated cross-cultural validity and reliability. Additionally, artificial neural network analysis is important for developing questionnaires and identifying the most influential standard indicators within the conceptual framework of the measured variables. Therefore, the focus is on measuring PR among children under 18 years of age to support the prevention and development of this age group, which represents society’s future and its strength.

## Figures and Tables

**Table 1:** Demographic characteristics of the study sample.

Sample	N	%	Gender		Mean Age	Std. Deviation
			Male	Female		
Saudi	440	42.27%	189	251	12.91	2.15
Egyptian	601	57.73%	264	337	12.97	2.141
Total	1041	100%	453	588	12.94	2.144

**Table 2:** Results of EFA of the PR questionnaire for the total sample (n = 1041).

Item	Loading	Item	Loading
Item 1: I enjoy exploring new experiences I’ve never had before.	0.919	Item 11: Trips and visits give me opportunities to learn and discover new things.	0.874
Item 2: I find enjoy in engaging in various adventures and activities from time to time.	0.888	Item 12: I reflect on the situations I experience to understand and learn from them.	0.864
Item 3: I love trying new things, no matter how challenging.	0.901	Item 13: I ask many questions when I want to understand things deeply.	0.857
Item 4: Visiting unfamiliar places ignites my enthusiasm.	0.889	Item 14: I enjoy thinking about things from multiple perspectives.	0.867
Item 5: I seek experiences that help me understand the world more deeply.	0.734	Item 15: I change my viewpoint when I hear a new and compelling idea.	0.863
Item 6: I draw valuable lessons from every situation I go through.	0.854	Item 16: I value discussion with those who disagree with me.	0.882
Item 7: I make sure to participate in activities that enrich my experience and broaden my horizons.	0.874	Item 17: I reflect on the events and situations I experience.	0.890
Item 8: I enjoy meeting new people and discovering their inspiring stories.	0.873	Item 18: I am moved by stories and events that prompt me to think deeply.	0.890
Item 9: I find pleasure in breaking the daily routine and trying different experiences.	0.863	Item 19: I am interested in understanding how others think and what makes them different from me.	0.880
Item 10: I feel that I grow when I have unfamiliar experiences.	0.886	Item 20: I write or draw to process my feelings and thoughts.	0.917

**Table 3:** Results of the EFA of the PR questionnaire for the Saudi sample (n = 440).

Item	Loading	Item	Loading
1	0.924	11	0.886
2	0.899	12	0.876
3	0.910	13	0.868
4	0.894	14	0.895
5	0.881	15	0.884
6	0.853	16	0.900
7	0.898	17	0.898
8	0.884	18	0.897
9	0.894	19	0.889
10	0.895	20	0.919

**Table 4:** Results of the EFA of the PR questionnaire for the Egyptian sample (n = 601).

Item	Factor 1	Factor 2
1	0.786	
2	0.848	
3	0.883	
4	0.831	
5	0.705	
6	0.769	
7	0.737	
8	0.774	
9	0.882	
10	0.783	
11	0.793	
12		0.867
13		0.890
14		0.882
15		0.714
16		0.763
17		0.766
18		0.827
19		0.865
20		0.738
Latin root	14.859	1.022
Explained variance	74.30%	5.10%

**Table 5:** Goodness-of-fitness indices for the general factor model and the two-factor model of the children’s PR questionnaire.

Model	Chi-Square		CFI		TLI		RMSEA		AIC		BIC	
	General	Two-Factors	General	Two-Factors	General	Two-Factor	General	Two-Factors	General	Two-Factors	General	Two-Factors
Saudi	1152.893	717.880	0.923	0.957	0.914	0.951	0.115	0.075	18,959.331	18,526.318	19,122.802	18,693.876
Egyptian	1797.06	663.741	0.894	0.968	0.881	0.964	0.126	0.07	27,978.756	26,847.432	28,154.700	27,027.775
Total	2726.005	1149.498	0.908	0.965	0.897	0.960	0.120	0.075	47,200.253	45,625.746	47,398.171	45,828.612

**Table 6:** Results of the CFA of the children’s PR two-factor model of questionnaire in the Saudi environment.

Factor	Item	Loading	Standard Error	Confidence 95%	
				Low	Upper
**Factor 1**	Item1	1.000	0.000	1.000	1.000
	Item2	0.916	0.027	0.863	0.968
	Item3	0.978	0.027	0.925	1.032
	Item4	0.920	0.027	0.866	0.973
	Item5	0.879	0.029	0.822	0.936
	Item6	0.808	0.029	0.752	0.864
	Item7	0.875	0.027	0.822	0.928
	Item8	0.886	0.027	0.833	0.940
	Item9	0.905	0.027	0.853	0.958
	Item10	0.900	0.027	0.846	0.954
	Item11	0.853	0.027	0.799	0.906
**Factor 2**	Item12	1.000	0.000	1.000	1.000
	Item13	0.995	0.037	0.923	1.067
	Item14	1.035	0.036	0.965	1.106
	Item15	1.023	0.036	0.952	1.094
	Item16	1.087	0.038	1.014	1.161
	Item17	1.082	0.037	1.011	1.154
	Item18	1.083	0.036	1.012	1.154
	Item19	1.073	0.037	1.001	1.145
	Item20	1.144	0.037	1.072	1.217

**Table 7:** Results of the CFA of the children’s PR two-factor model of questionnaire in the Egyptian environment.

Factor	Item	Loading	Standard Error	Confidence 95%	
				Low	Upper
**Factor 1**	Item1	1.000	0.000	1.000	1.000
	Item2	0.911	0.024	0.865	0.957
	Item3	0.956	0.023	0.911	1.002
	Item4	0.913	0.023	0.868	0.958
	Item5	0.869	0.045	0.782	0.956
	Item6	0.817	0.024	0.770	0.864
	Item7	0.797	0.024	0.751	0.843
	Item8	0.835	0.024	0.789	0.881
	Item9	0.791	0.024	0.745	0.837
	Item10	0.840	0.023	0.796	0.885
	Item11	0.831	0.023	0.785	0.877
**Factor 2**	Item12	1.000	0.000	1.000	1.000
	Item13	0.960	0.030	0.900	1.020
	Item14	0.945	0.031	0.886	1.005
	Item15	0.940	0.032	0.878	1.002
	Item16	1.046	0.033	0.982	1.110
	Item17	1.085	0.032	1.022	1.148
	Item18	1.115	0.032	1.052	1.179
	Item19	1.063	0.032	1.001	1.125
	Item20	1.114	0.031	1.054	1.174

**Table 8:** Results of the CFA of the children’s PR two-factor model of questionnaire in the total sample across cultures.

Factor	Item	Loading	Standard Error	Confidence 95%	
				Low	Upper
**Factor 1**	Item1	1.000	0.000	1.000	1.000
	Item2	0.915	0.018	0.881	0.950
	Item3	0.967	0.018	0.933	1.002
	Item4	0.915	0.018	0.881	0.950
	Item5	0.874	0.028	0.819	0.930
	Item6	0.814	0.018	0.778	0.850
	Item7	0.832	0.018	0.797	0.867
	Item8	0.860	0.018	0.824	0.895
	Item9	0.841	0.018	0.806	0.876
	Item10	0.868	0.018	0.833	0.902
	Item11	0.840	0.018	0.805	0.876
**Factor 2**	Item12	1.000	0.000	1.000	1.000
	Item13	0.976	0.023	0.929	1.022
	Item14	0.983	0.023	0.937	1.029
	Item15	0.975	0.024	0.928	1.021
	Item16	1.062	0.025	1.014	1.110
	Item17	1.085	0.024	1.038	1.133
	Item18	1.102	0.024	1.055	1.149
	Item19	1.068	0.024	1.021	1.114
	Item20	1.126	0.024	1.080	1.172

**Table 9:** Results of the centrality diagram analysis of the PR questionnaire for children in the Saudi environment.

Item	Betweenness	Closeness	Strength	Expected Influence
**R1**	10.000	0.005	1.382	1.144
**R10**	8.000	0.006	1.496	0.981
**R11**	10.000	0.006	1.700	0.865
**R12**	12.000	0.005	1.446	0.963
**R13**	3.000	0.005	1.279	0.905
**R14**	2.000	0.005	1.119	1.020
**R15**	13.000	0.005	1.571	0.898
**R16**	2.000	0.005	1.174	0.908
**R17**	6.000	0.005	1.285	1.027
**R18**	22.000	0.006	1.567	1.193
**R19**	3.000	0.005	1.036	0.949
**R2**	4.000	0.005	1.382	0.944
**R20**	2.000	0.004	1.002	0.904
**R3**	15.000	0.006	1.708	1.205
**R4**	5.000	0.005	1.413	1.002
**R5**	7.000	0.005	1.403	0.892
**R6**	1.000	0.005	1.393	0.824
**R7**	2.000	0.005	1.434	1.006
**R8**	25.000	0.007	1.848	0.933
**R9**	4.000	0.006	1.669	1.001

**Table 10:** Results of the centrality diagram analysis of items on the PR questionnaire for children in the Egyptian environment.

Item	Betweenness	Closeness	Strength	Expected Influence
**R1**	7.000	0.004	1.138	1.138
**R10**	14.000	0.005	1.360	1.007
**R11**	24.000	0.005	1.397	0.901
**R12**	9.000	0.004	1.213	0.898
**R13**	2.000	0.004	1.043	0.881
**R14**	17.000	0.005	1.440	0.971
**R15**	5.000	0.004	0.898	0.842
**R16**	2.000	0.004	1.040	0.914
**R17**	8.000	0.005	1.130	1.003
**R18**	3.000	0.004	1.199	1.128
**R19**	5.000	0.004	1.092	1.024
**R2**	9.000	0.004	1.132	1.012
**R20**	1.000	0.004	1.129	1.129
**R3**	9.000	0.004	1.219	1.131
**R4**	7.000	0.004	1.125	1.092
**R5**	15.000	0.005	1.197	0.924
**R6**	2.000	0.005	1.071	0.845
**R7**	2.000	0.004	1.076	0.878
**R8**	12.000	0.005	1.256	0.990
**R9**	9.000	0.005	1.254	0.805

**Table 11:** Results of the centrality diagram analysis of the items of the PR questionnaire for children in the total sample.

Item	Betweenness	Closeness	Strength	Expected Influence
**R1**	3.000	0.005	1.263	1.151
**R10**	17.000	0.006	1.528	0.996
**R11**	18.000	0.005	1.667	0.894
**R12**	10.000	0.005	1.352	0.925
**R13**	2.000	0.004	1.280	0.902
**R14**	4.000	0.005	1.466	1.011
**R15**	12.000	0.005	1.159	0.869
**R16**	2.000	0.004	1.260	0.903
**R17**	6.000	0.005	1.221	1.014
**R18**	11.000	0.005	1.386	1.149
**R19**	4.000	0.004	1.148	0.990
**R2**	5.000	0.004	1.325	0.972
**R20**	2.000	0.004	1.032	1.032
**R3**	9.000	0.004	1.481	1.177
**R4**	4.000	0.004	1.311	1.043
**R5**	5.000	0.005	1.466	0.904
**R6**	4.000	0.005	1.315	0.833
**R7**	8.000	0.005	1.516	0.955
**R8**	10.000	0.005	1.624	0.962
**R9**	6.000	0.005	1.646	0.891

**Table 12:** Reliability results of the children’s PR questionnaire in the Saudi, Egyptian, and total samples.

Sample	Cronbach’s α			ω McDonald		
	Factor 1	Factor 2	Total	Factor 1	Factor 2	Total
Saudi Arabia	0.978	0.975	0.987	0.979	0.975	0.988
Egypt	0.967	0.972	0.981	0.968	0.972	0.983
Total	0.972	0.973	0.983	0.973	0.973	0.985

**Table 13:** Descriptive statistics of differences in PR between the Saudi and Egyptian samples.

Variable	Groups	M	ST. Deviation	Bayesian Factor	Error %	Standard Error	Coefficient of Variance	95% Credible Interval	
								Lower	Upper
PR	Saudis	51.97	24.649	0.202	0.102	1.175	0.474	54.280	49.661
	Egyptians	54.18	23.545			0.960	0.435	56.066	52.294

## Data Availability

The data that support the findings of this study are available from the corresponding author upon reasonable request.
